# Pain management in preterm infants with necrotizing enterocolitis: an international expert consensus statement

**DOI:** 10.1007/s00431-025-06168-8

**Published:** 2025-05-14

**Authors:** Judith A. ten Barge, Gerbrich E. van den Bosch, Karel Allegaert, Aomesh Bhatt, Nicola Brindley, Dearbhla Byrne, Marsha Campbell-Yeo, Marta Camprubi-Camprubi, Giacomo Cavallaro, Xavier Durrmeyer, Nicholas Embleton, Mats Eriksson, Robert B. Flint, Felipe Garrido, Maria Lorella Giannì, Eric Giannoni, Heather Kitt, Daphne Klerk, Guðrún Kristjánsdóttir, Abigail Kusi Amponsah, Alexandre Lapillonne, Camilia R. Martin, Melinda Matyas, Elisabeth Norman, Shalini Ohja, Sofie Pirlotte, Ruth del Rio, Jean-Michel Roué, Catarina Sevivas, Rebeccah Slater, Anne Smits, Miguel Saenz de Pipaon, Manon Tauzin, Tiina Ukkonen, Sezin Unal, Eduardo Villamor, Eleanor J. Molloy, Sinno H. P. Simons

**Affiliations:** 1 Department of Neonatal and Pediatric Intensive Care, Division of Neonatology, Erasmus MC – Sophia Children’s Hospital, Rotterdam, The Netherlands; 2 Department of Development and Regeneration, KU Leuven, Leuven, Belgium; 3 Department of Pharmaceutical and Pharmacological Sciences, KU Leuven, Leuven, Belgium; 4 Department of Hospital Pharmacy, Erasmus Medical Center, Rotterdam, The Netherlands; 5 Department of Paediatrics, University of Oxford, Oxford, UK; 6 Department of Surgical Paediatrics, Royal Hospital For Sick Children, Glasgow, UK; 7 Discipline of Paediatrics, Trinity College Dublin, Dublin, Ireland; 8 School of Nursing, Faculty of Health, Dalhousie University, Halifax, NS Canada; 9 Department of Neonatology, BCNatal, Barcelona Center for Maternal Fetal and Neonatal Medicine, Hospital Sant Joan de Déu and Hospital Clinic, University of Barcelona, Barcelona, Spain; 10 Neonatal Intensive Care Unit, Fondazione IRCCS Ca’ Granda Ospedale Maggiore Policlinico, Milan, Italy; 11 Neonatal Intensive Care Unit, CHI Créteil, Créteil, France; 12 Université Paris Est Créteil, Faculté de Médecine de Créteil, IMRB, GRC CARMAS, Créteil, France; 13 Population Health Sciences Institute, Newcastle University, Newcastle Upon Tyne, UK; 14 Faculty of Medicine and Health, School of Health Sciences, Örebro University, Örebro, Sweden; 15 Department of Pediatrics, Clínica Universidad de Navarra, Madrid, Spain; 16 Department of Clinical Sciences and Community Health, Università Degli Studi Di Milano, Milan, Italy; 17 Clinic of Neonatology, Department Mother-Woman-Child, Lausanne University Hospital and University of Lausanne, Lausanne, Switzerland; 18 Division of Neonatology, Beatrix Children’s Hospital, University Medical Center Groningen, University of Groningen, Groningen, The Netherlands; 19 Faculty of Nursing and Midwifery, School of Health Sciences, University of Iceland, Reykjavik, Iceland; 20 Department of Public Health Nursing, School of Nursing and Midwifery, College of Health Sciences, Kwame Nkrumah University of Science and Technology, Kumasi, Ghana; 21 Department of Neonatology, Necker-Enfants Malades University Hospital, APHP, Paris, France; 22 Children’s Nutrition Research Center, Baylor College of Medicine, Houston, TX USA; 23 Division of Neonatology, Weill Cornell Medicine, New York, NY USA; 24 Department of Neonatology, Iuliu Hatieganu University of Medicine and Pharmacy, Cluj-Napoca, Romania; 25 Pediatrics, Department of Clinical Sciences Lund, Lund University, Skane University Hospital, Lund, Sweden; 26 Faculty of Medicine & Health Sciences, University of Nottingham, Nottingham, UK; 27 Neonatology, UZ Brussel, Jette, Belgium; 28 Department of Neonatology, Hospital Sant Joan de Déu, Institut de Recerca Sant Joan de Déu, Barcelona, Spain; 29 Department of Neonatal Medicine, University Hospital of Brest, Brest, France; 30 Neonatology, Clinical University Hospital of Santiago de Compostela, Health Research Institute of Santiago de Compostela (IDIS), RICORS, Santiago de Compostela, Spain; 31 Neonatal Intensive Care Unit, University Hospitals Leuven, Leuven, Belgium; 32 Neonatology, Instituto de Investigación Sanitaria del Hospital Universitario La Paz-IdiPAZ (La Paz University Hospital, Universidad Autónoma de Madrid), Madrid, Spain; 33 Research Unit of Clinical Medicine, and MRC Oulu, University of Oulu, Oulu, Finland; 34 Department of Pediatrics and Adolescent Medicine, Oulu University Hospital, Oulu, Finland; 35 Division of Neonatology, Baskent University Faculty of Medicine, Ankara, Turkey; 36 MosaKids Children’s Hospital, Maastricht University Medical Center (MUMC +), School for Oncology and Reproduction (GROW), Maastricht University, Maastricht, The Netherlands; 37 Trinity Translational Medicine Institute (TTMI), St James Hospital & Trinity Research in Childhood Centre (TRiCC), Dublin, Ireland; 38 Neurodisability, Children’s Hospital Ireland (CHI) at Tallaght, Dublin, Ireland; 39 Neonatology, CHI at Crumlin, Dublin, Ireland; 40 Paediatrics, Coombe Hospital, Dublin, Ireland

**Keywords:** Pain management, Preterm infants, Necrotizing enterocolitis

## Abstract

Necrotizing enterocolitis (NEC) is probably the most painful intestinal disease affecting infants born preterm. NEC is known to cause highly severe and prolonged pain that has been associated with adverse short- and long-term effects. However, research on pain management in infants with NEC is scarce. This is likely due to its low incidence and very acute occurrence. As a result, the optimal pain management for these vulnerable infants remains unknown, and analgesic therapy practices are highly variable. Therefore, we aimed to establish expert-based consensus recommendations on pain management for NEC. Experts of the European Society for Paediatric Research (ESPR) Special Interest Groups on Neonatal pain and NEC were invited to participate in two consensus meetings. Prior to the first hybrid consensus meeting, an online survey provided input for potential recommendations. During the consensus meetings, experts shared clinical expertise and voted on recommendations. An expert consensus statement, comprising nine recommendations on optimal pain assessment and pain treatment in infants with NEC, was developed. Expert recommendations included regular pain assessments with a neonatal pain scale with additional assessments on indication and pre-emptive administration of analgesic therapy (e.g., paracetamol and an opioid) in infants with NEC stage ≥ II.

*Conclusion*: This expert consensus statement provides clinical recommendations essential for any healthcare professional caring for premature infants with NEC. The recommended guidance this statement provides on pain management strategies is key to preventing and reducing pain in this vulnerable population.

**Table:** 

**What is Known:** *• Necrotizing enterocolitis (NEC) is a very painful disease, making effective pain management essential.* • *Current pain management practices for infants with NEC are highly variable.*	
**What is New:** *• This expert consensus statement provides recommendations on optimal pain assessment and pain treatment in infants with NEC.* • *These clinical recommendations may help better prevent pain in these vulnerable infants.*

**Supplementary Information:**

The online version contains supplementary material available at 10.1007/s00431-025-06168-8.

## Introduction

Necrotizing enterocolitis (NEC) is a severe intestinal and systemic disease predominantly affecting very preterm infants, with an incidence of approximately 5 to 10% in very low birth weight infants and causing severe morbidity and mortality [1, 2]. While the etiology is not fully understood, NEC is characterized by inflammation and ischemia in the intestines, most commonly the ileum, often leading to severe sepsis with respiratory and circulatory insufficiency [3, 4]. Its treatment includes bowel rest (nil by mouth (NBM)/nil per os (NPO)) and antibiotics, and in severe cases, infants require hemodynamic support and surgical intervention to resect the affected part of the intestines. The condition induces both acute procedural and prolonged visceral pain [5–7], making effective pain management an essential pillar in NEC treatment.

NEC is considered one of the most painful conditions in neonatal medicine. Untreated pain can disrupt normal physiology, including neuronal development. This can lead to short-term clinical instability as well as long-term consequences such as reduced physical growth, poor development, and long-term cognitive and behavioral impairments [8, 9]. Despite this, research to identify optimal pain management in infants with NEC is very limited. A recent survey among European neonatologists highlighted a paucity of research identifying the most effective analgesic therapy regimen for these infants, meaning that analgesic therapy practices vary widely [10]. Infants with NEC are often very preterm and critically ill, making them especially vulnerable for the negative effects of pain as well as for the potential adverse effects of analgesics. A recent survey identified that many neonatologists would find guidelines helpful in improving pain management for infants with NEC [10]. Therefore, we organized consensus meetings among international experts in neonatal pain and NEC to develop recommendations for pain management in infants with NEC.

## Methods

Experts from the European Society for Paediatric Research (ESPR) Special Interest Groups (SIGs) for neonatal pain and necrotizing enterocolitis were invited to participate in two consensus meetings. These SIGs include experts from across the globe (20 countries, including countries outside Europe) and various disciplines, including pain researchers, neonatologists, nurses, and pharmacists.

A brief online survey (LimeSurvey GmbH, Hamburg, Germany) was shared with members of the two SIGs prior to the first consensus meeting to gather input on potential recommendations. This anonymous survey included questions about what pain assessment and analgesic therapy in infants with NEC should entail, including pain measurement instruments, pain assessment frequency, non-pharmacological interventions, and analgesic therapy (Supplementary material 1), and was designed based on the results of the previous European survey regarding pain management in NEC [10]. Those who designed and analyzed the preparatory survey (JB, GB, and SS) did not participate in the voting or discussion during the consensus meetings.

The first consensus meeting was held on October 18, 2024, during the European Academy of Paediatric Societies (EAPS) congress in Vienna. Participants could join in person or online. The survey results were presented during this meeting, and participants were encouraged to share their insights and experiences. Proposed statements were voted on anonymously using a five-point Likert scale ranging from strongly disagree to strongly agree using Mentimeter (Mentimeter AB, Stockholm, Sweden). Statements for which the combined “agree” and “strongly agree” votes exceeded 80% were included in the final consensus statement. Given the diverse range of topics of the statements and the differing expertise of participants, participants were requested not to vote on statements for which they had no opinion.

Statements that did not meet this threshold were adapted based on participant feedback. The revised statements were discussed and voted on during a second, fully online meeting on November 19, 2024, along with statements that were not addressed in the initial meeting due to time constraints. Two statements that did not reach consensus during the second meeting were voted on via an additional online survey.

In January and February 2025, draft versions of the expert consensus statement were distributed among consensus meeting participants and revised based on their feedback. All participants approved the final version of the consensus statement.

## Results

Twenty-three experts completed the preparatory survey for the consensus meeting, of whom 14 were members of the SIG for neonatal pain, 6 of the SIG for necrotizing enterocolitis, and 3 of both SIGs. Twenty-nine experts participated in the first consensus meeting and 26 in the second consensus meeting (Supplementary material 2). The final expert consensus statement contains nine recommendations for pain assessment and pain treatment in infants with NEC (Table 1).

**Table 1:** Recommendations for pain management in infants with NEC

Recommendation		Agreement
*Pain assessment*		
1	Pain should be assessed with a neonatal pain scale	100%
2	Pain should be assessed at least 6 times per 24 h	91%
3	Indications for additional pain assessments are: patient shows signs of pain; follow-up after a high pain score; analgesic therapy has been changed (initiated, increased, or decreased)	100%
4	When available, pain monitoring technologies could be considered in addition to observational pain scores	86%
*Pain treatment*		
5	Before painful procedures in infants with NEC, sucrose should be considered	94%
6	Skin-to-skin care could be considered in infants with NEC, depending on the infant’s cardiorespiratory condition	87%
7	Pre-emptive analgesic therapy should be initiated in NEC stage ≥ II, and pre-emptive analgesic therapy should be considered in NEC stage I	83%
8	As initial analgesic therapy, paracetamol and an opioid could be considered	82%
9*	If initial analgesic therapy is insufficient despite maximum dosing, one should… • If initial analgesic therapy includes morphine: consider switching to a stronger opioid** • If initial analgesic therapy includes a stronger opioid**: consider adding a sedative	81%

*There was no consensus on whether to start with morphine or a stronger opioid as a first-choice opioid. Therefore, two strategies for intensified analgesic therapy are suggested, depending on the chosen opioid for initial analgesic therapy. If initial analgesic therapy only includes paracetamol (in case of mild/stage I NEC), the first step would be to add an opioid

**Defined as synthetic opioids with a higher potency than morphine, including fentanyl, sufentanil, and remifentanil

### Pain assessment

During the first round of voting, a strong consensus was reached on *the importance of assessing pain using a neonatal pain scale* (statement 1) and *conducting a minimum of six pain assessments per day* (statement 2). There was no clear consensus about the type of pain assessment scale to use. Participants considered scale usability, NICU staff experience, validity (for prolonged pain and age-appropriateness), and reliability as important factors determining the choice of pain scale.

Discussions arose regarding the indications for additional pain assessments, particularly the need for pain scoring during painful procedures. There was no consensus about using pain assessment tools during care-related procedures. Participants mentioned that for procedures known to be painful, appropriate (non-) pharmacological treatment should be provided, with adjustments during the procedure if signs of pain are observed (using a procedural pain score). After removing painful procedures from the list of indications, consensus was reached in the second round of voting: *patient shows signs of pain, follow-up after a high pain score, or analgesic therapy has been changed (initiated, increased, or decreased)* (statement 3).

Moreover, there was debate about using additional pain monitoring technologies, such as heart rate variability and skin conductance monitors. Some participants felt these techniques were insufficiently validated, while others considered these techniques a useful addition given the limitations of current pain scales in infants with NEC. Participants disagreed with the original statement that these technologies should be considered when available. After revising the statement to “could” rather than “should,” agreement was reached during a second round of voting: *When available, pain monitoring technologies could be considered in addition to observational pain scores* (statement 4).

### Pain treatment

#### Non-pharmacological

Participants agreed on considering administration of sucrose (or another oral sweet tasting solution, e.g., 24% sucrose or 30% glucose) during painful procedures in infants with NEC during the first round of voting: *Before painful procedures in infants with NEC, sucrose should be considered* (statement 5). However, participants commented that large amounts of sucrose could be contra-indicated as infants with NEC are on an NPO regimen. Other potential non-pharmacological methods of pain relief, such as skin-to-skin care (kangaroo care), can be considered, but depending on the condition of the infant: *Skin-to-skin care could be considered in infants with NEC, depending on the infant’s cardiorespiratory condition* (statement 6). Participants commented that a multimodal approach to non-pharmacological pain management may be worth considering, for example, combining sweet tasting solutions with non-nutritive sucking and containment.

#### Pharmacological

Participants agreed on the pre-emptive administration of analgesic therapy (i.e., before the infant starts showing signs of pain) in infants with NEC stage II or higher: *Pre-emptive analgesic therapy should be initiated in NEC stage* ≥ *II and considered in NEC stage I* (statement 7). However, the discussion highlighted the variability in pain levels among infants with NEC stage I (where NEC diagnosis might still be doubted or only suspected) as well as variability in Bell’s staging.

Regarding the choice of agents for initial analgesic therapy, most participants supported the inclusion of paracetamol (acetaminophen), but there was disagreement over the use of the word “should” as paracetamol may be contra-indicated in specific cases. Moreover, some participants raised concerns regarding potential unknown long-term adverse effects of paracetamol, though others mentioned that equal concerns apply to other drugs and that insufficiently treated pain is known to have detrimental short- and long-term effects. No consensus was reached on whether the initial analgesic regimen should differ between NEC stage II and stage III or on the choice between morphine and stronger opioids (defined as synthetic opioids with a higher potency than morphine, including fentanyl, sufentanil, and remifentanil).

Participants who favored fentanyl mentioned advantages such as quicker onset, lack of active metabolites, effectiveness, and favorable adverse effect profile (less hypotension) compared with morphine. Those who favored morphine pointed to its extensive use and experience in preterm infants, stronger evidence for safety, and lower risk of tolerance compared with fentanyl. Regardless of the choice, some participants suggested rotating opioids after approximately 1 week to prevent tolerance.

No consensus emerged on whether to start with a high analgesic dose and decrease it when possible or to begin with a low dose and increase as needed. However, participants agreed that dosing should be titrated based on pain levels and patient-specific characteristics (e.g., higher dose postoperatively).

**Fig. 1:**
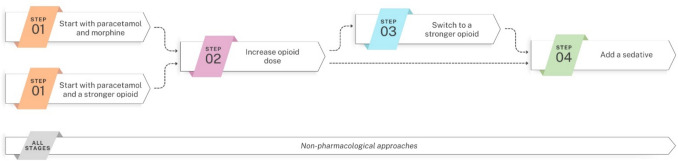
Suggested steps in analgesic therapy for infants with NEC. Non-pharmacological approaches should be considered in all stages

If initial analgesic therapy proved inadequate, participants recommended a stepwise approach: *firstly increasing the opioid dosage, then switching to a stronger opioid if one had not already been used, and finally adding a sedative* (statement 9, Fig. 1). Preferred agents with sedative effects, ranked in descending order of popularity, included dexmedetomidine, midazolam, and (es)ketamine. The lack of evidence on safety was noted as a challenge in supporting one sedative over another. Additionally, participants commented that the choice of sedative is influenced by the infant’s gestational age.

## Discussion and conclusion

Effective pain management for infants in the NICU is crucial, not only for ensuring their comfort but also for mitigating potentially detrimental short-term and long-term consequences on neurodevelopment and other critical developmental outcomes [11]. However, research into optimal pain management for NEC, one of the most painful conditions in neonatal care, has been lacking, and current analgesic therapy practices for NEC are highly variable [10]. This variability in current practices also offers an opportunity to learn from each other. By convening an international group of experts in neonatal pain and NEC, we attempted to reach a consensus on recommendations for pain management in NEC. The resulting expert consensus statement provides practical guidance on both pain assessment and pain treatment in infants with NEC.

Though there was strong consensus on the need to regularly assess pain with a neonatal pain scale, there was no clear consensus on the choice of pain scale. The chosen pain scale should preferably be multidimensional or, at least, cover multiple domains (e.g., facial expressions and movements) [12]. Experts emphasized the importance of using a scale validated for assessing prolonged pain in preterm infants, recommending the use of a scale familiar to those working in the NICU. None of the available pain scales has been validated in infants with NEC. During the development of the Échelle Douleur Inconfort Nouveau-né (EDIN) scale, Debillon et al. examined pain behaviors during the first days of NEC [13]. They found that rather than classical pain behaviors, infants with NEC commonly displayed a blank face and few movements, putting them at risk of unrecognized pain. Therefore, the EDIN scale incorporated a blank face and infrequent movements as possible signs of pain. Other commonly used scales for prolonged pain in infants are the COMFORTneo scale and the Neonatal Pain, Agitation, and Sedation Scale (N-PASS). Validation studies for the COMFORTneo and N-PASS also included infants with NEC, though these studies did not describe pain behaviors during NEC separately [14, 15]. Regardless of NEC status, pain assessment can be challenging in extremely preterm infants due to dampened pain behaviors [16, 17]. For procedural pain assessment, the Premature Infant Pain Profile (-Revised) (PIPP(-R)) is one of the most well-established pain scales [18–20]. Infants with NEC experience both procedural pain and prolonged pain, which require assessment with different pain scales. Future studies are needed to examine the validity of existing pain scales in infants with NEC and the potential benefits of incorporating pain monitoring technologies like heart rate variability and skin conductance monitoring [21].

Though non-pharmacological interventions form the basis of pain management in the NICU, there is no evidence on the use of non-pharmacological techniques during NEC. Oral sweet solutions (sucrose or glucose) are among the most extensively researched interventions for procedural pain in infants [22–24]. However, their use has not been studied specifically in infants with NEC, and fewer than half of NICUs use sucrose for these infants, likely because they are on a nil per os (NPO) regimen [10, 25]. Although the analgesic effects of sucrose rely on oral ingestion and taste mechanisms [26], sucrose is absorbed intestinally and would therefore theoretically be contra-indicated in infants with NEC [27]. However, sucrose solutions as small as 0.1 mL have been shown to reduce pain scores [28], and such small volumes may be acceptable during an NPO regimen. Similarly, small volumes of oropharyngeal colostrum/breast milk may be used for pain relief, as well as immunological benefits in infants with NEC [29]. Another non-pharmacological technique with well-established value in infants—for procedural pain management and infant-parent bonding—is skin-to-skin care (kangaroo care) [30]. However, skin-to-skin care has not been studied in infants with NEC. Previous World Health Organization (WHO) recommendations for care in preterm infants recommended initiating continuous skin-to-skin care only after the infant had been stabilized [31]. However, recent studies have shown that skin-to-skin care is feasible even in unstable infants [32–34], and that immediate skin-to-skin care after birth lowers mortality and improves cardiorespiratory stability [35, 36], prompting a change in the WHO recommendations [37]. As infants with NEC are often critically ill, individual assessment of the safety and feasibility of skin-to-skin care is required. Moreover, research is needed to determine the effectiveness and safety of non-pharmacological pain management strategies during NEC. Animal studies suggest that skin-to-skin care may reduce NEC disease pathology [38].

To prevent pain and its potentially harmful effects, pre-emptive analgesic therapy is provided to infants at high risk of experiencing pain, such as postoperatively. This also applies to infants with confirmed NEC. However, NEC diagnosis is challenging and Bell’s staging has been criticized [39]. Especially, NEC stage I is highly variable, eliciting discussion about balancing risks and benefits of pre-emptive analgesic therapy in these infants, as well as about which analgesics to administer. To mitigate risks, analgesic therapy should be titrated based on pain scores. Evidence regarding the effectiveness of different analgesics in infants with NEC is very scarce, with only two previous studies describing pain management during NEC [5, 6]. These studies found that infants with NEC required high doses of morphine and that some infants nonetheless experienced episodes of (persistent) pain [5, 6]. Though this suggests morphine may be insufficient to treat pain during NEC, it is unknown if other analgesics are superior. Statements 8 and 9, on the choices of initial and intensified analgesic therapy, elicited much discussion among participants, and there was no consensus on whether to choose morphine or a stronger opioid. In infants with NEC, fentanyl may have a more favorable adverse effects profile, as it has been associated with less gastrointestinal dysmotility and hemodynamic instability [40, 41]. However, fentanyl has been associated with greater tolerance [41]. To avoid tolerance, opioid rotation may be used [42]. Moreover, discussion arose regarding the safety of paracetamol [43]. Additionally, preclinical research found that prostaglandin E2 improved intestinal perfusion in experimental NEC [44], suggesting COX inhibition by paracetamol (or NSAIDs) may be harmful during NEC. Considering the current uncertainty and lack of robust evidence on the effectiveness of different analgesics during NEC, final recommendations for analgesic therapy during NEC were phrased as suggestions rather than definitive guidelines. Moreover, the lack of a formal literature search is a limitation of this statement.

**Fig. 2:**
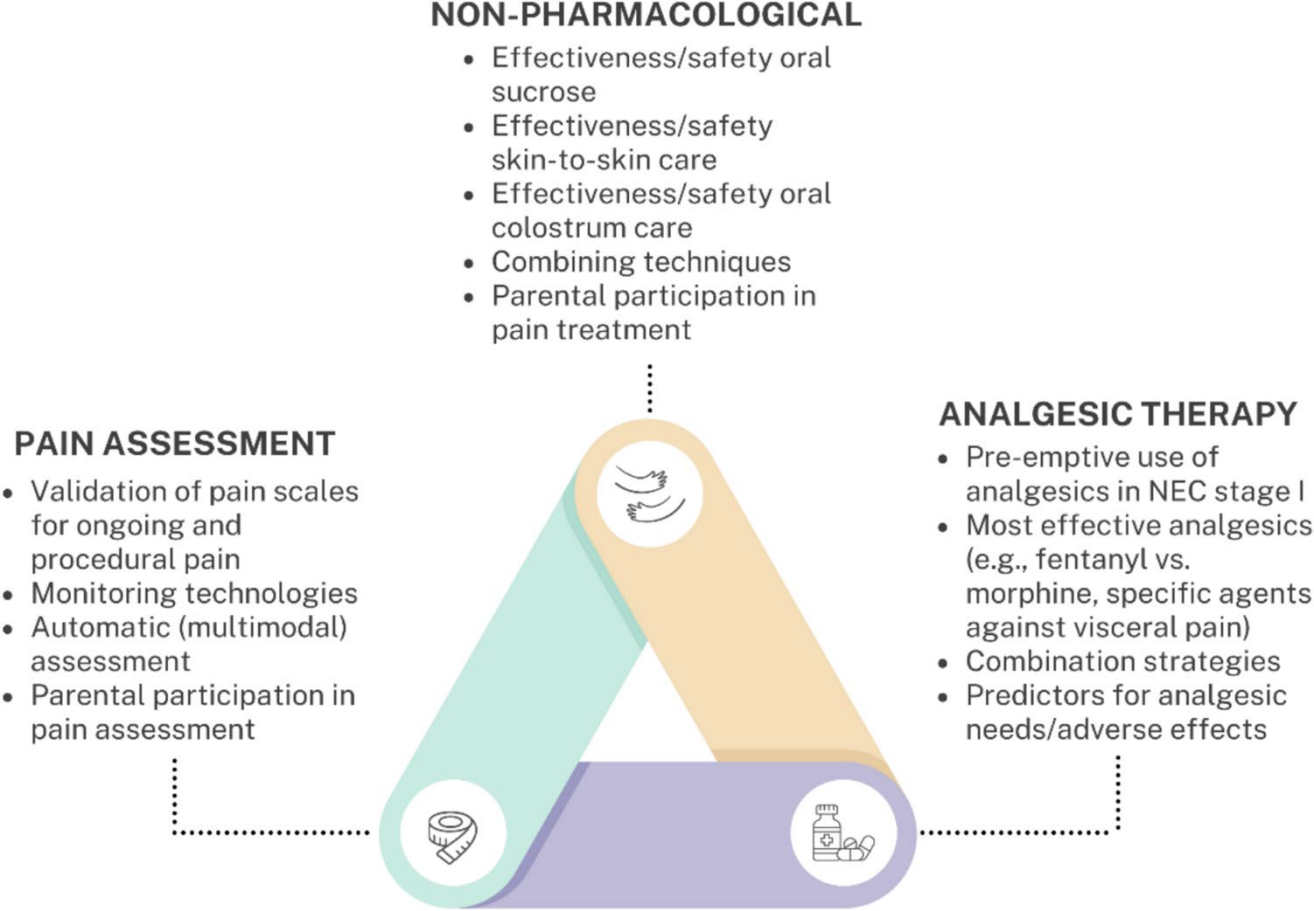
Research agenda for improving pain management in infants with NEC

This expert consensus statement intends to guide NICU clinicians by offering evidence- and expertise-informed recommendations, while acknowledging the need for flexibility based on individual patient characteristics and NICU context. Ensuring medication safety in neonates is challenging due to the vulnerability of this population, leading to poor information quality regarding pharmacokinetics, adverse reactions, and long-term consequences. These recommendations could be used to adapt or create local pain management protocols. Furthermore, this statement aims to stimulate further research into optimal pain management for infants with NEC, including the effectiveness and required doses of different opioids as well as novel agents specifically targeting visceral pain [45]. Figure 2 proposes a research agenda for pain management in infants with NEC, based on current knowledge gaps identified by the authors. Large, multicenter studies are needed to obtain definitive answers regarding optimal pain management in infants with NEC, as many NICUs treat no more than ten infants with NEC yearly [10]. Future studies could use a prospective observational design, for instance, through a registry, to increase feasibility. In the meantime, the utilization of best available evidence and clinical expertise, as integrated in this consensus statement, remains vital.

## Supplementary Information

Below is the link to the electronic supplementary material.

Supplementary file1 (PDF 123 KB)

## Data Availability

No datasets were generated or analysed during the current study.

## Abbreviations

NEC: Necrotizing enterocolitis
NBM: Nil by mouth
NPO: Nil per os
ESPR: European Society for Paediatric Research
SIG: Special Interest Group
EAPS: European Academy of Paediatric Societies
NICU: Neonatal Intensive Care Unit
N-PASS: Neonatal Pain, Agitation, and Sedation Scale
EDIN: Échelle Douleur Inconfort Nouveau-né
PIPP(-R): Premature Infant Pain Profile (-Revised)
WHO: World Health Organization
COX: Cyclooxygenase
NSAIDs: Non-steroidal anti-Inflammatory drugs
