# Supplementary material for: Pain management in preterm infants with necrotizing enterocolitis: an international expert consensus statement
Source: Eur J Pediatr. 2025 May 14;184(6):342. doi: 10.1007/s00431-025-06168-8 (PMC12078406; doi:10.1007/s00431-025-06168-8)
Supplement: Supplementary file 1 — Supplementary file1 (PDF 123 KB) [file 431_2025_6168_MOESM1_ESM.pdf]

Contents

Supplementary material 1. Overview of the questionnaire 1

Supplementary material 2. Participants in the consensus meetings 3

Supplementary material 1. Overview of the questionnaire

Question	Question type	Answer options
**Demographics**		
Where do you work?	OQ	Free text
What is your function?	OQ	Free text
Of which SIG are you a member?	MC	• SIG for Neonatal Pain<br>• SIG for Necrotizing Enterocolitis<br>• Both<br>• Neither
**Pain assessment**		
Which pain scale(s) should be used in infants with NEC?	CQ	• COMFORTneo score<br>• Numerical Rating Scale (NRS)<br>• Neonatal Infant Pain Scale (NIPS)<br>• Neonatal Pain, Agitation, and Sedation Scale (N-PASS)<br>• Premature Infant Pain Profile (-Revised) (PIPP(-R))<br>• Échelle Douleur Inconfort Nouveau-né (EDIN)<br>• Cries, Requires oxygen, Increased vital signs, Expression, Sleeplessness (CRIES)<br>• Neonatal Facial Coding System (NFCS)<br>• Bernese Pain Scale for Neonates (BPSN)<br>• ALPS-Neo<br>• Douleur Aiguë Nouveau-né (DAN) scale<br>• Face, Legs, Activity, Cry, and Consolability (FLACC)<br>• Neonatal Infant Acute Pain Assessment Scale (NIAPAS)<br>• Leuven Neonatal Pain Score (LNPS)<br>• Other: …
How often should pain at least be assessed in infants with NEC (regular pain assessments)?	OQ	[Free text] times per day (24 h)
When should additional pain assessments be performed (on indication)?	CQ	• Patient shows signs of pain<br>• Patient previously exhibited a high pain score<br>• Analgesic therapy has recently been initiated

<table>
<tr><td></td><td></td><td>

Analgesic dose has recently been increased
Analgesic therapy has recently been decreased or discontinued
During painful procedures
Other: …

</td></tr>
<tr><td>Should monitoring technologies (e.g., heart rate variability) (also) be used to assess pain in infants with NEC? If yes, please specify which technologies and when.</td><td>MC + OQ</td><td>

Yes
No
I don’t know

Free text</td></tr>
<tr><td>Analgesic therapy</td><td></td><td></td></tr>
<tr><td>When should analgesic therapy be initiated in infants with NEC?</td><td>CQ</td><td>

Pre-emptively: NEC stage I or higher
Pre-emptively: NEC stage IIa or higher
Pre-emptively: NEC stage IIb or higher
Pre-emptively: NEC stage IIIa or higher
Pre-emptively: NEC stage IIIb or higher
Pre-emptively: perioperative
Only on indication: when the patient starts showing signs of pain
Other: …

</td></tr>
<tr><td>Which agent(s) should initial analgesic therapy (step 1) include?</td><td>CQ</td><td>

Paracetamol (acetaminophen)
Morphine
Fentanyl
Sufentanil
Remifentanil
Methadone
Oxycodone
Midazolam
Ketamine
Dexmedetomidine
Other:

</td></tr>
<tr><td>How should analgesic therapy be intensified if initial analgesic therapy is not sufficient?
You may choose one strategy (i.e., increase the dose, add agent(s) or switch) for each step. Please fill in which agent should be increased, added or switched in the corresponding box.</td><td>CQ</td><td>Free text
<table>
<tr><th></th><th>Increase dose of…</th><th>Add…</th><th>Switch from … to …</th></tr>
<tr><td>Step 2</td><td></td><td></td><td></td></tr>
<tr><td>Step 3</td><td></td><td></td><td></td></tr>
<tr><td>Step 4</td><td></td><td></td><td></td></tr>
<tr><td>Step 5</td><td></td><td></td><td></td></tr>
<tr><td>Step 6</td><td></td><td></td><td></td></tr>
</table>
</td></tr>
<tr><td>Based on which parameters should analgesic therapy in infants with NEC be decreased?</td><td>OQ</td><td>Free text</td></tr>
<tr><td>Should sucrose be used for procedural pain in infants with NEC?
You can provide a reason for your choice in the comment box.</td><td>MC + OQ</td><td>

Yes
No

Free text</td></tr>
</table>

Which non-pharmacological interventions should be used for pain management in infants with NEC? And which non-pharmacological interventions should be avoided?	OQ	Should be used in infants with NEC: [Free text]<br>Should be avoided in infants with NEC: [Free text]
**Comments**		
Do you have any additional suggestions or comments?	OQ	Free text
**Abbreviations: MC = multiple choice; CQ = checkbox question (i.e. multiple options can be chosen); OQ = open question**		

Supplementary material 2. Participants in the consensus meetings

Meeting 1 (hybrid)

Sinno Simons (The Netherlands)
Judith ten Barge (The Netherlands)
Robert Flint (The Netherlands)
Marta Camprubi-Camprubi (Spain)
Janno Schouten (The Netherlands)
Kelly Storm (The Netherlands)
Ruth del Rio (Spain)
Giacomo Cavallaro (Italy)
Eduardo Villamor (The Netherlands)
Felipe Garrido (Spain)
Melinda Matyas (Romania)
Eleanor Molloy (Ireland)
Miguel Saenz de Pipaon (Spain)
Heather Kitt (United Kingdom)
Rebeccah Slater (United Kingdom)
Aomesh Bhatt (United Kingdom)
Daphne Klerk (The Netherlands)
Manon Tauzin (France)
Shalini Ohja (United Kingdom)
Anne Smits (Belgium)
Jean-Michel Roué (France)
Catarina Sevivas (Spain)
Gerbrich van den Bosch (The Netherlands)
Abigail Kusi Amponsah (Ghana)
Elisabeth Norman (Sweden)
Guðrún Kristjánsdóttir (Iceland)
Tiina Ukkonen (Finland)
Xavier Durrmeyer (France)
Mats Eriksson (Sweden)

Meeting 2 (online)

Judith ten Barge (The Netherlands)
Sinno Simons (The Netherlands)
Robert Flint (The Netherlands)
Gerbrich van den Bosch (The Netherlands)
Karel Allegaert (Beglium)
Alexandre Lapillonne (France)
Nicola Brindley (United Kingdom)
Sofie Pirlotte (Belgium)
Maria Lorella Giannì (Italy)
Rebeccah Slater (United Kingdom)
Heather Kitt (United Kingdom)
Miguel Saenz de Pipaon (Spain)
Daphne Klerk (The Netherlands)
Giacomo Cavallaro (Italy)
Nicholas Embleton (United Kingdom)
Eric Giannoni (Switzerland)
Elisabeth Norman (Sweden)
Marsha Campbell-Yeo (Canada)
Dearbhla Byrne (Ireland)
Catarina Sevivas (Spain)
Tiina Ukkonen (Finland)
Sezin Unal (Turkey)
Guðrún Kristjánsdóttir (Iceland)
Camilia Martin (United States)
Anne Smits (Belgium)
Eleanor Molloy (Ireland)
